# Use of existing systematic reviews for the development of evidence-based vaccination recommendations: Guidance from the SYSVAC expert panel

**DOI:** 10.1016/j.vaccine.2023.02.027

**Published:** 2023-03-17

**Authors:** Antonia Pilic, Sarah Reda, Catherine L. Jo, Helen Burchett, Magdalena Bastías, Pauline Campbell, Deepa Gamage, Louise Henaff, Benjamin Kagina, Wiebe Külper-Schiek, Carole Lunny, Melanie Marti, Rudzani Muloiwa, Dawid Pieper, James Thomas, Matthew C. Tunis, Zane Younger, Ole Wichmann, Thomas Harder

**Affiliations:** a Robert Koch Institute, Seestrasse 10, 13353 Berlin, Germany; b Faculty of Public Health & Policy, London School of Hygiene and Tropical Medicine (LSHTM), 15-17 Tavistock Place, London WC1H 9SH, United Kingdom; c Independent Consultant, Santiago, Chile; d Nursing, Midwifery and Allied Health Professions Research Unit, Glasgow Caledonian University, Govan Mbeki Building, Glasgow G4 0BA, United Kingdom; e Epidemiology Unit and Advisory Committee on Communicable Diseases, Ministry of Health, #231, De Saram Place, Colombo 10, Sri Lanka; f World Health Organization, Avenue Appia 20, 1211 Geneva, Switzerland; g University of Cape Town, Faculty of Health Sciences, Observatory, 7925 Cape Town, South Africa; h Knowledge Translation Program, St Michael’s Hospital, Unity Health Toronto, and Cochrane Hypertension Review Group, University of British Columbia, 2176 Health Sciences Mall, Vancouver, BC V6T1Z2, Canada; i Brandenburg Medical School Theodor Fontane, Faculty of Health Sciences Brandenburg, Institute for Health Services and Health System Research, 15562 Rüdersdorf bei Berlin, Germany; j Evidence for Policy and Practice Information and Co-ordinating (EPPI-) Centre, UCL Social Research Institute, University College London, 10 Woburn Square, London WC1H 0NR, United Kingdom; k Public Health Agency of Canada, Centre for Immunization Readiness, 130 Colonnade Road, A.L. 6501H, Ottawa, Ontario K1A 0K9, Canada; l Brandenburg Medical School Theodor Fontane, Center for Health Services Research, 15562 Rüdersdorf bei Berlin, Germany

**Keywords:** Evidence-based medicine, Immunization recommendation, Methodology, Systematic review, Vaccination

## Abstract

National immunization technical advisory groups (NITAGs) develop immunization-related recommendations and assist policy-makers in making evidence informed decisions. Systematic reviews (SRs) that summarize the available evidence on a specific topic are a valuable source of evidence in the development of such recommendations. However, conducting SRs requires significant human, time, and financial resources, which many NITAGs lack. Given that SRs already exist for many immunization-related topics, and to prevent duplication and overlap of reviews, a more practical approach may be for NITAGs to use existing SRs. Nevertheless, it can be challenging to identify relevant SRs, to select one SR from among multiple SRs, or to critically assess and effectively use them.

To support NITAGs, the London School of Hygiene and Tropical Medicine, Robert Koch Institute and collaborators developed the SYSVAC project, which consists of an online registry of systematic reviews on immunization-related topics and an e-learning course, that supports the use of them (both freely accessible at https://www.nitag-resource.org/sysvac-systematic-reviews). Drawing from the e-learning course and recommendations from an expert panel, this paper outlines methods for using existing systematic reviews when making immunization-related recommendations. With specific examples and reference to the SYSVAC registry and other resources, it offers guidance on locating existing systematic reviews; assessing their relevance to a research question, up-to-dateness, and methodological quality and/or risk of bias; and considering the transferability and applicability of their findings to other populations or settings.

## 1 Introduction

### 1.1 Background

National immunization technical advisory groups (NITAGs) are panels of experts that provide evidence-based recommendations to policy-makers and program managers in their countries on immunization-related issues [1]. They hail from a variety of disciplines, including clinical medicine, epidemiology, infectious diseases, public health, immunology, clinical research, health economics, health systems, and social sciences. The work of NITAGs is ideally supported by a technical secretariat and funded predominantly by government and/or partner organizations [1]. Despite this support, NITAGs, particularly in less resourced countries, are often pressed for technical resources in evidence reviewing when fulfilling their mandate.

NITAGs draw on empirical evidence when developing the recommendations for vaccination policies in their respective country. Systematic reviews (SRs) are a useful evidence source, particularly for questions on the benefits and harms of interventions as on vaccine efficacy, effectiveness, duration of protection, and safety. SRs provide a comprehensive summary of the evidence in a given area [2]. They use systematic and transparent methods to identify all studies that are potentially relevant to a research question, select studies for inclusion, appraise the quality of included studies, and synthesize study results. By compiling available data, they provide more precise estimates of intervention effects than single studies [3], [4], yet they can vary considerably in the quality and standard of SRs. **Appendix 1,** Table S1 presents an overview of different types of reviews to distinguish SRs from other types of reviews and evidence syntheses.

Historically, NITAGs have conducted or commissioned SRs on similar areas in the field of vaccination, increasing the potential for duplication and overlap [5]. To prevent duplication and given the significant time, training, and expertise needed to conduct *de novo* SRs, a more efficient use of NITAGs’ limited resources may be to make greater use of already existing SRs. If a high-quality SR for the question of interest exists, NITAGs may be able to focus their time and efforts on applying the findings of the existing SR to their local contexts and gathering other policy-relevant information (e.g., stakeholder input) [3], [4]. However, it is not always easy to identify existing SRs that match a decision-maker’s research question or to select one SR from among multiple SRs.

The specific objectives are (a) to highlight relevant resources and tools for using existing SRs and (b) to propose considerations to be taken into account when searching for and using existing SRs. Key factors to consider are summarized in flowcharts that can be used as tools to identify which SR may be favored. The paper is not intended to be a comprehensive guide to using existing SRs (e.g., using data from *meta*-analyses or extraction tables) or to replace established or mandated processes of evidence-based decision-making (e.g., GRADE (Grading of Recommendations Assessment, Development and Evaluation)). The intended audiences are NITAG members, NITAG Secretariats, and experts globally involved in the development of recommendations on the vaccination and immunization policies. This guidance and the examples focus on SRs of quantitative studies, particularly those investigating the efficacy, effectiveness, and safety of vaccines. However, the outlined steps can be applied also to SRs of qualitative studies as well, for example, on topics such as vaccine coverage and administration.

The optimal process for reaching the best evidence-based recommendations may vary from country to country and local settings.

### 1.2 Development of this guidance

In December 2019, the Robert Koch Institute (RKI) hosted an International Experts Workshop on “Methods for Using Systematic Reviews” [6], during which an international expert panel of immunization experts and methodologists of NITAGs and their secretariats (Australia, Canada, Chile, China, Germany, South Africa, Sri Lanka, USA), multilateral organizations (World Health Organization (WHO), ECDC), and academia (Glasgow Caledonian University, London School of Hygiene and Tropical Medicine (LSHTM), University College London, University of British Colombia, University of Cape Town, Witten/Herdecke University) shared experiences and reached consensus on methods for using SRs. Results of the workshop informed the further development of the SYSVAC registry, originally developed by the LSHTM and hosted by WHO [5], and establishment of the accompanying e-learning course on the use of existing SRs when developing recommendations (both now freely available at https://www.nitag-resource.org/sysvac-systematic-reviews). The SYSVAC registry aims to facilitate NITAGs’ retrieval of SRs by compiling SRs on immunization-related topics and enabling filtering by country, region, disease/pathogen, publication date, and target population. It additionally provides methodological quality assessments for SRs and other information that may aid NITAGs in their selection of SRs to use (e.g., date of last literature search, number of studies). The e-learning course includes exercises and handouts intended to help NITAGs deepen and refresh their knowledge on using SRs. This paper summarizes recommendations of the SYSVAC expert panel and highlights how NITAGs can use the SYSVAC online registry in the recommendation-making process.

## 2 Steps for using existing systematic reviews

The following six steps provide guidance on the use of existing SRs in the development of evidence-based vaccination recommendations. There are six main steps for using existing SRs [7], which can be applied to the vaccination field [8] and are similar to frameworks leveraged in other health technology assessment areas [9].

1) Defining PICO (Population, Intervention, Comparison, Outcome) elements,
2) locating existing SRs,
3) assessing their relevance and up-to-dateness,
4) assessing methodological quality and/or risk of bias,
5) determining appropriate use and incorporating the results of existing SRs, and
6) assessing applicability and transferability to local context

Each of the steps will be discussed in further detail below.

### 2.1 Defining PICO (Population, Intervention, Comparison, Outcome) elements

The recommendation-making process usually starts with a broad policy question, which may be posed by a Ministry of Health and directed to the NITAG or developed by the NITAG itself [10]. To facilitate the search, screening, and analysis of SRs, this broad policy question should be refined and structured to a specific one. Specific policy questions, around which NITAGs develop a vaccination recommendation should include a population, intervention (and comparison group), and outcome or goal of the recommendation (see **Appendix A2**). These elements are known as PICO (Population, Intervention, Comparison, Outcome) [11].

See Table 1 for examples of different types of policy questions. As can be seen with the PCV13 (pneumococcal conjugate vaccine) example, a broad policy question may be broken down into multiple specific policy questions to address different facets. A taxonomy of types of overlap in PICO criteria was developed [12] to help authors map the PICO to different SRs on the same topic. The taxonomy of overlap can help NITAGs assess the relevance of their specific policy question to that of the existing SRs.

**Table 1:** Examples of broad policy questions, specific policy questions, and PICO elements.

**Broad policy question**	**Specific policy question (including population, intervention, and outcome or goal)**	**P**opulation **I**ntervention **C**omparison **O**utcome (e.g. on benefits and harms of interventions)
Should routine HPV vaccination of female adolescents be recommended?	Should routine HPV vaccination, with two doses of any HPV vaccine given at least 5 months apart, be recommended in 9- to 14-year-old girls to reduce HPV infections and HPV-associated cancers?	P: 9–14-year-old girls I: 2 doses of HPV vaccination C: No vaccination O: Efficacy/effectiveness against HPV infection, anogenital warts/condyloma, cervical, oropharyngeal, anal and vaginal/vulvar (pre)cancer
Should influenza vaccination be recommended for pregnant women?	Should a single dose of any influenza vaccine be recommended for pregnant women in the 2nd trimester to reduce influenza-related hospitalization during pregnancy?	P: Pregnant women in 2nd trimester I: Single dose of influenza vaccine C: No vaccination O: Efficacy/effectiveness against hospitalization, premature birth, acute respiratory, cardiopulmonary, pneumonia and influenza diseases
Should PCV13 be recommended for infants in routine immunization programs?	a) Should three doses of PCV13 be administered routinely to immunocompetent infants aged < 12 months to reduce the overall incidence of invasive pneumococcal disease?	P: Immunocompetent infants aged < 12 months I: 3 doses of PCV13 C: No vaccination O: Invasive pneumococcal disease
	b) Given the risk of serious adverse events after vaccination, should immunocompetent infants < 12 months of age receive any dose of PCV13 vaccine to induce protection against pneumococcal disease?	P: Immunocompetent infants aged < 12 months I: Any dose of PCV13 C: No vaccination O: Serious adverse events
	c) Should immunocompetent infants < 12 months of age be given three instead of four doses of PCV13 to reduce the number of severe pneumococcal diseases?	P: Immunocompetent infants aged < 12 months I: 3 doses of PCV13 C: 4 doses of PCV13 O: Invasive pneumococcal disease, pneumococcal community-acquired pneumonia, hospitalizations due to pneumococcal disease, and deaths

HPV: *human papillomavirus*; PCV: pneumococcal conjugate vaccine.

### 2.2 Locating existing systematic reviews

When searching for existing SRs, NITAGs should take a systematic, transparent, and reproducible approach. Such an approach entails identifying databases to search, defining eligibility criteria, and reviewing search results.

#### 2.2.1 Identifying databases to search

To ensure a comprehensive search for SRs, it is recommended to search both databases that exclusively or mostly contain SRs and general bibliographic databases that include reviews among other publications [7], [13]. An empirical study identified that 99.2 % of SRs on health-related topics were found by searching MEDLINE, Epistemonikos, and reference checking [14].

*The SYSVAC registry includes reviews from MEDLINE, Embase, and the Cochrane Database of Systematic Reviews. SYSVAC includes SRs, including living, umbrella, and, for COVID-19 related topics, rapid reviews*. Thus, if NITAGs are searching for non-systematic reviews (e.g., narrative reviews) or primary studies in their vaccination recommendation process, their search of SYSVAC should be supplemented with other bibliographic databases, as well as the grey literature and reference checking. Refer to **Appendix A2** for details on relevant databases to search and on devising a search strategy.

#### 2.2.2 Defining eligibility criteria

Criteria for including or excluding SRs need to be pre-defined to provide the framework for reviewing the evidence. Inclusion criteria should include the PICO elements and other outcomes of interests (see Table 2). There may be SRs that do not fit the NITAG’s PICO precisely but rather are narrower or broader in scope. For example, when reviewing evidence on the efficacy of influenza vaccination during pregnancy, there might be relevant information both in SRs involving only pregnant women and in SRs involving a broader population, such as healthy adults.

**Table 2:** Example of eligibility criteria.

NITAGs should decide how to operationalize the concept of a ‘systematic review.’ For example, they may decide to include only Cochrane Reviews, generally viewed as gold standard, which are prepared and supervised by a Cochrane Review Group and updated to reflect the findings of new evidence [15]. SRs included in the SYSVAC registry are determined to be systematic in nature if the reviews label themselves as SRs in the title or abstract of the paper or describe the following minimum eligibility criteria such as those used by Robinson and colleagues [16]:

1) Explicit and adequate search
2) Application of pre-specified eligibility criteria
3) Assessment of quality or risk of bias of included studies
4) Synthesis or attempted synthesis of results

An exclusion criterion to consider is industry sponsorship. Previous studies have reported that private industry-sponsored randomized controlled trials and SRs with *meta*-analysis are more likely to report intervention-favourable results compared to other sources of funding [17], [18], [19]. Industry funded groups may undertake advocacy, education and research activities that echo their sponsors’ interests. For these reasons, some NITAGs may not be comfortable basing policy decisions on SRs led by vaccine manufacturers due to potential sponsorship bias. It is advisable to determine whether industry sponsorship would preclude study eligibility [20].

#### 2.2.3 Reviewing search results and deciding how to proceed

**Fig. 1:**
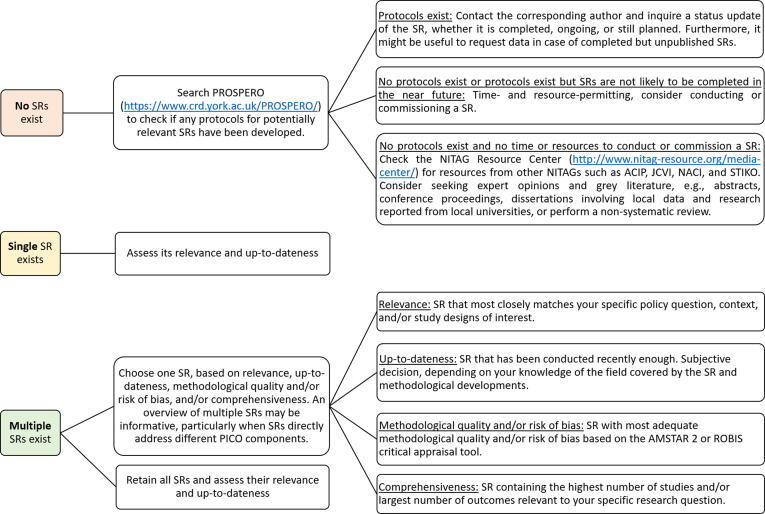
Scenarios for proceeding after reviewing search results. ACIP: Advisory Committee on Immunization Practices (NITAG United States of America); AMSTAR 2: A Measurement Tool to Assess systematic Reviews 2; JCVI: Joint Committee on Vaccination and Immunization (NITAG United Kingdom); NACI: National Advisory Committee on Immunization (NITAG Canada); PROSPERO: International prospective register of systematic reviews; SR: systematic review; ROBIS: Risk Of Bias In Systematic reviews; STIKO: Ständige Impfkommission (NITAG Germany).

Different procedures can be followed depending on whether no SR was found, only one SR was found or multiple SRs exist (see Fig. 1).

In the event that multiple SRs appear in the list of search results, NITAGs can consider choosing just one SR to use based on its relevance, up-to-dateness, methodological quality and/or risk of bias, or comprehensiveness (see Fig. 1). In practice, this decision may be challenging, and rarely will the most relevant SR also be the most up-to-date, of highest quality, and most comprehensive. Thus, NITAGs should be prepared to consider which criteria are most pertinent to the country context and be aware that prioritizing one criterion may mean neglecting another. Decision rules can be created *a priori*, e.g., if there are multiple SRs of the same methodological quality and/or risk of bias, the SR including the most studies for the outcome of interest will be selected first, followed by the most recent SR.

Another option at this juncture is to retain multiple SRs and proceed with assessing their relevance and up-to-dateness (see **2.3**). NITAGs could later consider reviewing the results of all relevant, up-to-date SRs of sufficient quality informally or by conducting an overview of SRs [13], [21], [22]. This approach would be particularly appropriate in cases where several SRs address different components of the PICO (e.g., SRs on different outcomes or populations within the PICO).

### 2.3 Assessing relevance and up-to-dateness

After locating SRs, it is important that NITAGs assess them for relevance and up-to-dateness, as the SR(s) informing a recommendation should be as closely relevant to the research question and as up-to-date as possible.

**Fig. 2:**
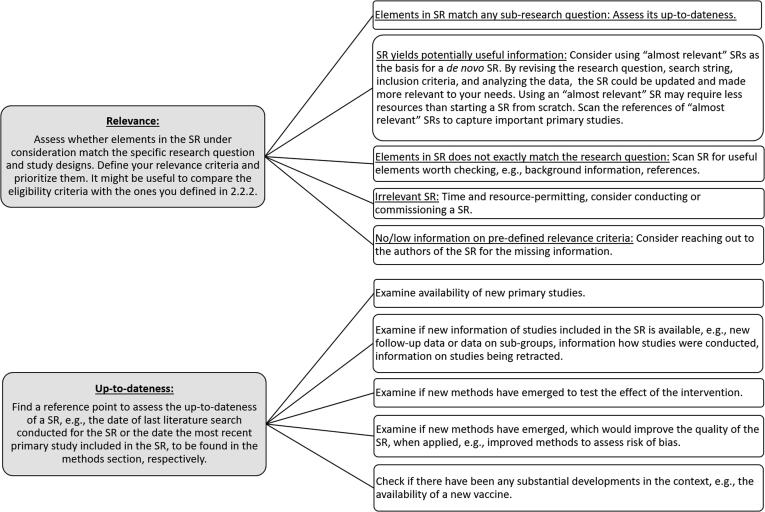
Aspects to consider when assessing relevance and up-to-dateness [7], [16], [23]. SR: systematic review.

Relevance is the extent to which an existing SR matches the specific question, the context (e.g., setting, time frame), and/or study designs considered. Up-to-dateness, sometimes referred to as “currency,” is the extent to which a SR has been conducted recently enough to meet the needs of NITAGs. Mostly based on the available guidance for relevance [7], [12], [16] and up-to-dateness [23], Fig. 2 outlines factors that may assist with this determination. **Appendix A3** contains a concrete example on how to assess the relevance and up-to-dateness of a SR related to vaccination.

Deciding whether a SR is “up-to-date” can be difficult, as it is a subjective decision dependent on knowledge of the field covered by the SR. Regardless of the criteria used to determine up-to-dateness, the methods section of the SR, specifically, the date of last literature search conducted for the SR or the date of the most recent primary study included in the SR plays a critical role in this assessment. These dates serve as reference points for the decision on whether new relevant studies, methods, or information have emerged. *The SYSVAC registry highlights the date of last literature search for its indexed SRs.*

Detailed guidance on how to update SRs goes beyond the scope of this paper. However, it is worth pointing out that, regardless of the reason for updating the SR (see **Appendix 4,** Figure S1 for examples), each update should begin with a “protocol refresh,” or a review of the background, research question, inclusion criteria, and methods of the existing SR [23]. This process will help updaters think through all the aspects of a SR which may need to be updated and plan their update accordingly. It will also help them document their process, which is important for future updating efforts and for transparency. Comparing the protocol of an update to that of an existing SR can provide clues as to why findings across the SRs might differ. Additional resources and guidance on updating SRs can be found elsewhere [9], [23], [24].

#### 2.3.1 Outcome of assessments of relevance and up-to-dateness

**Fig. 3:**
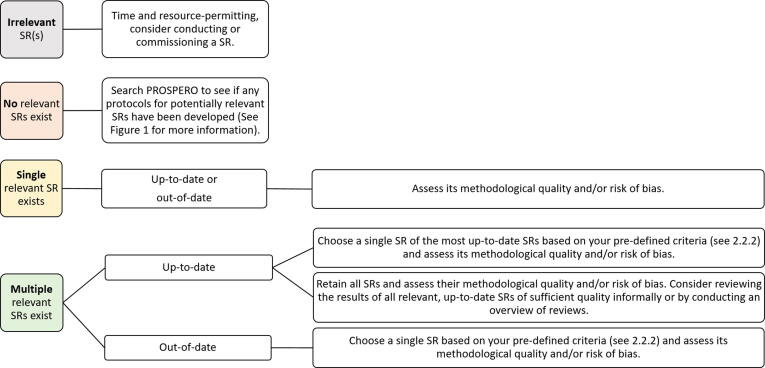
Scenarios for proceeding after assessing relevance and up-to-dateness. SR: systematic review.

NITAGs might encounter different results when assessing the relevance and up-to-dateness of SRs. Potential scenarios of relevance and up-to-dateness are shown in Fig. 3. On the right-hand side of the figure, approaches for proceeding in a given situation are described.

### 2.4 Assessing methodological quality and/or risk of bias

SRs should be used as the basis for reviewing evidence in the vaccination recommendation process only if they meet certain quality or risk of bias ratings. This step explains the concepts of quality and risk of bias and ways of assessing them.

#### 2.4.1 Methodological quality, risk of bias, and systematic review reporting

Methodological quality refers to how well the SR is conducted according to established guidance (e.g., Cochrane Handbook for Systematic Reviews of Interventions [27], JBI Manual for Evidence Synthesis [28]). SRs with low methodological quality have flaws that have an uncertain impact on review findings [29].

Risk of bias refers to the extent to which systematic flaws or limitations in the design, conduct, or analysis of a SR influence the results or conclusions [30]. Methodological quality and risk of bias are usually inversely related, such that SRs of high methodological quality also generally have low risk of bias. Nonetheless, it is possible for a SR to have poor methodological quality and be at low risk of bias, e.g., if a SR reports and discusses all methodological shortcomings according to the assessment tool guidelines but still contain fatal flaws, such as an insufficiently comprehensive search strategy leading to missing information [31].

SR reporting refers to the extent to which authors of SRs clearly and adequately report their approach and findings in their published manuscript. Clear and comprehensive reporting is necessary for the assessment of methodological quality and risk of bias. Without it, assessors of SRs cannot reliably interpret the findings of the SR, understand its strengths and limitations, or compare it with other SRs. To improve reporting of SRs and *meta*-analyses and provide a transparent checklist which SR authors can use to report their methods and findings, guidelines like PRISMA (Peer Review of Electronic Search Strategies) [25] and the MOOSE guide (meta-analyses Of Observational Studies in Epidemiology) [26] have been developed. PRISMA focuses on SRs of studies evaluating the effects of health interventions, irrespective of the design of the included studies, while MOOSE focuses on SRs of non-randomized studies. Both may also be useful for critical appraisal of published SRs although it is not a quality assessment instrument to gauge the quality of a SR[25].

#### 2.4.2 Tools to assess methodological quality and/or risk of bias of systematic reviews

A commonly used tool to assess the methodological quality of a SR is AMSTAR 2 (A MeaSurement Tool to Assess systematic Reviews 2) [29]. ROBIS (Risk Of Bias In Systematic reviews) aims to assess the risk of bias of a SR [30]. Both tools are appropriate for appraising SRs of randomized and non-randomized studies and have significant overlap. However, they differ in some items, the time required to apply them to a SR (“scoring time”), and intended area(s) of focus (see Table 3). For example, conflict of interest, which is increasingly relevant in the vaccine review literature, is assessed by AMSTAR 2 but not by the ROBIS tool. Therefore, NITAGs using ROBIS might consider assessing conflict of interest as an additional step (e.g., determine whether a SR was authored by vaccine industry employees). While AMSTAR 2 has a narrower focus on SRs on healthcare interventions [32], ROBIS was designed to address a wider variety of research questions, including interventions, diagnosis, prognosis, and etiology [29], [30].

**Table 3:** Comparison of AMSTAR 2 and ROBIS.

**Name of tool**	**Concept measured**	**Areas of research**	**Number of items**	**Items unique to tool (and associated item number)**	**Type of overall rating(s)**	**Characteristics of users**	**Scoring time** (from [34])
AMSTAR 2 [29]	Methodological quality	SR of healthcare interventions	16 items	- List of excluded studies and justification for exclusion provided by authors (7) - Sources of funding of included studies reported (10) - Potential conflicts of interests reported (16)	Overall confidence in the results of the review (critically low/ low/moderate/high), based on assessments of critical domains, pre-determined by user (appraisers can alternatively use the critical domains suggested by the AMSTAR 2 developers)	Health professionals and policy makers. No advanced training in epidemiology required.	20 min ± 12 min*
ROBIS [30]	Risk of bias	SR of interventions, diagnosis, prognosis, and etiology	21 items – 4 key domains, each including 5–6 signaling questions and a field for concerns	- Review relevant to research question (Phase 1, optional) - Appropriate eligibility criteria for research question (1.2) - Appropriate restrictions on information sources (1.5) - Relevant study results collected for synthesis (3.3) - Data possibly missing from synthesis (4.1) - Adherence to protocol (4.2)	Risk of bias ratings assigned for each domain and overall (low/high/unclear)	Authors of overviews of SRs, guidance developers, reviewers who may want to assess risk of bias in their SR once it is complete or to minimize the risk of bias when planning review methods. Content and methodological expertise needed.	29 min ± 17 min*

AMSTAR 2: A MeaSurement Tool to Assess systematic Reviews 2; ROBIS: Risk Of Bias In Systematic reviews; SR: systematic review.

*time to complete the assessment.

It is worth noting that AMSTAR 2 and ROBIS exhibit a few notable limitations [31]. As both critical appraisal tools are largely based on expert opinion, much work is to be done to establish their reliability and validity. Furthermore, they depend on high reporting quality comprehensiveness. Assessments are based on how methods and results are reported in SRs, so if reporting quality comprehensiveness is low, neither AMSTAR 2 nor ROBIS can provide valid assessments. Neither tool can capture content-related flaws, as they cannot assess whether the research question under consideration is weak or irrelevant, or determine if there are any relevant studies missing or incorrect data extraction that can lead to large differences in pooled effect estimates in *meta*-analyses [33]. Ideally, only a content expert should decide these issues.

A concrete example of how AMSTAR 2 can be applied to a SR related to vaccination is shown in **Appendix A4**, Table S5.

NITAGs relying on existing SRs could assess either the methodological quality or risk of bias of reviews, or both. The key point is that some sort of critical assessment is necessary, using a valid and reliable instrument. A study by Pieper and colleagues suggested that reliability was slightly higher for AMSTAR 2 that for ROBIS, yet there was a high correlation between both tools, suggesting validity [31]. Regardless of the tool chosen, it is important that its use by NITAGs be piloted on a sample of SRs, particularly when multiple people will be assessing the quality and/or risk of bias of existing SRs independently and in duplicate [31]. Pilot testing helps ensure that assessments are being performed consistently and increase inter-rater reliability [35]. *SYSVAC users could consider using the AMSTAR 2 assessments provided in the registry, which have been performed by trained SYSVAC staff.*

#### 2.4.3 Results of methodological quality and/or risk of bias assessment

Determining whether a SR’s quality assessment results are “good enough” for a NITAG to use the review in the recommendation-making process is a challenge. One approach is to decide on a cut-off result or range. For example, SRs with “moderate” or “high” overall quality ratings might be used, based on their performance along the seven critical domains in AMSTAR 2. If using ROBIS, SRs with a “low” overall risk of bias rating might be used.

**Fig. 4:**
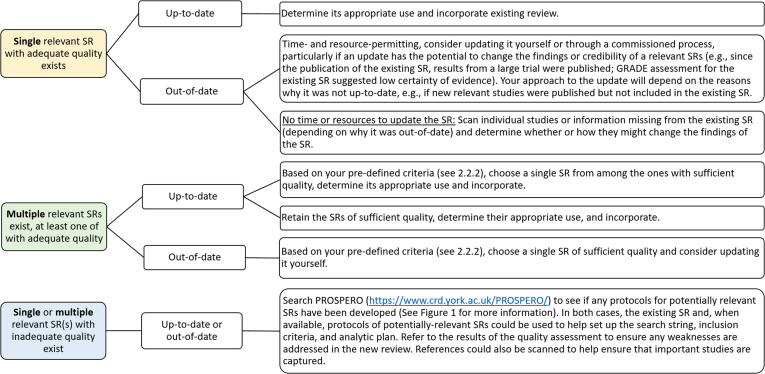
Scenarios on how to proceed based on methodological quality and/or risk of bias assessments. For conciseness, “methodological quality and/or risk of bias” will be referred to as “quality.” SR: systematic review.

Possible scenarios and corresponding approaches for proceeding in a given situation are described in Fig. 4.

### 2.5 Determining appropriate use and incorporating existing reviews

At this point in the process, NITAGs have either identified a single SR or multiple SRs to use in the evidence review process. These SRs should be relevant to the NITAG’s specific policy question, up-to-date, and of sufficient quality/risk of bias. The following section describes how NITAGs might determine which elements to incorporate into the evidence review process. While the focus is on the use of a single SR, some guidance and resources are provided in the event NITAGs wish to use multiple SRs.

#### 2.5.1 Considerations when using systematic review(s)

When using an existing relevant and up-to-date SR of sufficient quality, the following elements of the SR (see below) might be incorporated into the evidence review:

1) Data extraction – to check for quality of data abstraction, or if taking a narrower scope than the existing SR
2) Study-level quality/risk of bias assessment – to check standard of quality appraisal, to contrast quality appraisal of different SRs, or if quality criteria different to that used in the SR, or if taking a narrower scope
3) Synthesis – to combine and evaluate the extracted data to determine the outcomes of the existing SR

*When using a single systematic review*

(1) Data extraction

Data extraction tables of existing SR should be examined carefully to determine if they meet respective needs and quality standards. For example, only tables where at least two reviewers performed data extraction and where results of individual trials are reported should be used. Any missing information across the SR should be extracted from the original included primary studies or contacting the authors of the SR or authors of the included studies [7]. The data extracted by authors of the existing SR and data gleaned from SR or authors of included studies should be clearly distinguished, e.g., by using separate tables. If primary studies will be included in the evidence review that are not already in the existing SR, they need to be distinguished as well [16].

(2) Study-level quality/risk of bias assessment

Primary studies for all relevant outcomes should be assessed for quality/risk of bias. When deciding whether to use existing quality assessments, consider the following points:

• Have the authors of existing SR reported their process of critically appraising included studies clearly and was an accepted and up-to-date critical appraisal tool used (e.g., ROBINS-I for non-randomized studies of interventions [32], RoB 2 for randomized controlled trials (Risk of Bias 2) [36])?
• If including primary studies in the evidence review in addition to those in the existing SR, is the critical appraisal tool used in the existing SR similar to the tool used for the other primary studies and performed similarly?

If the conditions to any of the above points or questions are not met, consider not using existing assessments and instead redo the quality/risk of bias assessment, using an accepted tool of choice.

(3) Synthesis

The results of a single SR should be integrated if its methods are in line with those used by the NITAG or NITAG Secretariat for finding evidence, assessing quality of included studies, and grading the strength of evidence. If primary studies are included in the evidence review in addition to those in the existing SR, distinguish them, both narratively and in tables.

*When using multiple systematic reviews*

When facing multiple relevant, up-to-date, high-quality existing SRs addressing the immunization policy question, either the results of all can be reviewed informally or an overview of SRs can be conducted. An overview of SRs synthesizes evidence from multiple SRs. It may investigate the same intervention for different conditions, problems, or populations; multiple outcomes of the same intervention for the same condition or population; or adverse effects from a single intervention or class of related interventions for one or more conditions [37]. While overviews of reviews resemble SRs in the use of systematic, transparent, and reproducible methods, a key distinction is that their unit of searching, inclusion, and analysis is SRs, as opposed to primary studies [38]. Further information on conducting overviews of SRs can be found in the literature [13], [21], [22].

However, general considerations when facing multiple SRs are mentioned below. The focus is on the use of existing data abstraction, study-level quality/risk of bias assessments, and syntheses since these elements are likely to be the most relevant when integrating multiple SRs in an evidence review.

(1) Data extraction

Consider the same points as for the use of data extraction tables from a single SR (see above).

(2) Study-level quality/risk of bias assessment

When deciding whether to use existing quality assessments, consider the same points as for the use of an existing quality assessment from a single SR (see above), in addition to the following questions:

• Did existing SRs use the same tool for critical assessment?
• Were quality/risk of bias assessments performed consistently and correctly across existing SRs? NITAGs can assess this by quality-checking a sample of studies across the SRs and/or extracting all quality/risk of bias assessment data, comparing them across SRs, and recording discrepancies [22]. Any missing information necessary for assessing the studies or for reconciling discrepancies across assessments can be added by referring to included studies or contacting the authors of the reviews or authors of the included studies.

Quality/risk of bias assessments should be added if missing for any of the SRs. Redoing quality/risk of bias assessments for individual studies across multiple SRs can result in significant amount of work and expenses, if the NITAG does not have access to the full text of studies. The feasibility of such an undertaking should be considered. *The SYSVAC registry informs users about whether or not the SRs included in the registry are available as open access.*

(3) Synthesis

When integrating the results of multiple SRs, it is important to describe each SR, including findings, number and types of included studies, number of study population, and point estimates of effect measures and their confidence intervals [16]. These elements can generally be taken from the SRs, although quality checks should be performed to ensure that one agrees with how the SR authors synthesized their data and to prevent errors in data extraction. New analyses may be necessary if the SRs analyze different populations or subgroups [7], if existing SRs are to be supplemented with new primary studies or with new or different *meta*-analytic methods, or if the results of the existing SRs are not trustworthy [22].

An overlap (or lack of overlap) in included primary studies across the SRs should be shown visually [39], [40]. This can be done by using a matrix like in Table 4 below, where the grey boxes represent the primary studies in each SR. By scanning the rows, it becomes visible which of the studies are included in all SRs. Alternatively, to facilitate the assessment of overlap of primary studies among SRs, free tools like GROOVE (Graphical Representation of Overlap for OVErviews) [41] might be used.

**Table 4:** Sample matrix to present overlapping studies across systematic reviews.

Grey boxes represent the primary studies in each systematic review.

A common challenge in synthesizing multiple existing SRs is that they may reach different results and/or conclusions, based on different PICO, methodological decisions, judgments, or interpretations made by the authors [42], [43]. To address this challenge, the following non-mutually-exclusive approaches should be considered:

1) Determine reasons for the discordance and discuss these in the report [7].
2) Present the discordance in a table or graphic [22].
3) Use a decision rule, which summarizes the process for identifying and resolving causes of discordance, like the Jadad algorithm [42], to select just one SR.

### 2.6 Assessing applicability and transferability

The following section provides initial considerations regarding the applicability and transferability of evidence from identified SRs to a specific setting for which a vaccination recommendation will be developed.

Applicability is the extent to which an intervention can be implemented in a new setting or how feasible it is to implement an intervention elsewhere [44]. Transferability is the extent to which the effectiveness of an intervention achieved in one setting can be achieved in another [44]. Both concepts are important to consider in evidence-based decision making, given the context-specific nature of public health interventions. An intervention that was effective in one setting might be ineffective in another, due to contextual factors.

#### 2.6.1 Checklist of contextual factors

Table 5 offers a preliminary list of contextual factors to consider when thinking about how evidence from SRs might work in a specific population or setting. To identify characteristics stratifying health opportunities and outcomes, consider using the PROGRESS-Plus framework (Place of residence, Race/ethnicity/culture/language, Occupation, Gender/sex, Religion, Education, Socioeconomic status, Social capital) [45], [46], [47] and other factors adapted to the vaccine context [48], [49]. Not all of these factors will be relevant to every SR that is being used by NITAGs and may be the optimal method for applicability assessments [50]. Decision-makers might prioritize these factors in different ways.

**Table 5:** Checklist of contextual factors to consider how evidence from systematic reviews might work in a specific population or setting.

**Setting and population** Consider the setting and population for which you are developing vaccination recommendations.	• How similar is your setting to the settings of the individual studies included in the SR? Consider the available financial and human resources and existing services, policies, and programs.
	• How similar is your population to the study population? Consider, e.g., sociodemographic profile, beliefs, values, immunological conditions, host genetics, use of medications, prior exposure to or vaccination against similar viruses, likelihood of coinfection with other pathogens, geographic factors, epidemiological factors (including local burden of disease), literacy, and maternal health/breastfeeding context (i.e., to assess exposure to maternal antibodies and antigens in breastmilk). Also consider the heterogeneity/homogeneity of the study populations compared to your population.
	• How easily could the intervention be implemented in your setting? Consider potential facilitators and barriers. These may be related to the availability of resources, skills of local staff, organizational factors, and social and political environment (e.g., acceptability, political will).
**Intervention** Consider the intervention examined in the SR and compare it to what is locally available or are considered locally.	• What were the intervention components (e.g., vaccine, vaccine components)?
	• Who delivered the intervention?
	• How was the intervention implemented (e.g., dose schedule)?
	• What resources (e.g., financial, human, equipment) were required to deliver the intervention?
	• Was the intervention delivered as intended? Was it adapted or modified over time?
**Outcomes** Consider the outcomes examined in the SR and compare them to outcomes relevant to your context.	• What unintended effects or adverse events were reported? Did these vary by population subgroup or intervention approach?
	• What diagnostic tests (e.g., assay) were used?

SR: systematic review.

## 3 Conclusions

In practice, it will not always be easy to identify existing SRs that match a decision-maker’s research question or to select one SR from among multiple SRs. Relevance, up-to-dateness, methodological quality and/or risk of bias, and comprehensiveness are all important criteria, and rarely will the most relevant SR also be the most up-to-date, of highest quality, and most comprehensive. Thus, NITAGs should be prepared to reconsider key criteria and make tradeoffs when using SRs for the development of vaccination recommendations.

Additional tools that may assist NITAGs in using SRs are the e-learning course and SYSVAC registry, which are both freely accessible at https://www.nitag-resource.org/sysvac-systematic-reviews.

## Declaration of Competing Interest

The authors declare that they have no known competing financial interests or personal relationships that could have appeared to influence the work reported in this paper.

## Data Availability

No data was used for the research described in the article.
