# Supplementary material for: Use of existing systematic reviews for the development of evidence-based vaccination recommendations: Guidance from the SYSVAC expert panel
Source: Vaccine. 2023 Mar 17;41(12):1968–78. doi: 10.1016/j.vaccine.2023.02.027 (PMC10015272; doi:10.1016/j.vaccine.2023.02.027)
Supplement: Supplementary data 1 [file mmc1.docx]

**Appendix**

**Use of existing systematic reviews in the development of evidence-based vaccination recommendations: Guidance from the SYSVAC expert panel**

# Appendix A1) Introduction

## Types of reviews

**Table S1**. Review types characterized by methods used; adapted [1-4]

| **Type of review** | **Description** | **Search** | **Appraisal** | **Synthesis** | **Analysis** |
| --- | --- | --- | --- | --- | --- |
| Literature review (narrative, non-systematic review) | Generic term to describe an examination of published literature | May or may not be comprehensive | Usually does not include quality or risk of bias assessment | Typically narrative | May be chronological, thematic, conceptual, etc. |
| Systematic review | Compilation of evidence based on the systematic search, selection, appraisal, and synthesis of primary studies and adherence to a pre-specified protocol | Aims to be exhaustive and comprehensive | Includes quality or risk of bias assessment. Assessment may determine inclusion/exclusion. | Typically narrative with tables | What is known and unknown about a topic, recommendations for practice and future research, certainty of findings and interpretation in light of possible biases |
| Meta-analysis | Method that statistically combines the results of quantitative primary studies to provide a more precise effect of the results. Can be conducted within a systematic review. | When conducted within a systematic review, aims to be exhaustive and comprehensive. May use funnel plot to assess completeness/publication bias. | Includes quality or risk of bias assessment. Assessment may determine inclusion/exclusion and/or sensitivity analyses. | Graphical and tabular with narrative commentary | Numerical analysis of measures of effect assuming absence of heterogeneity |
| Network meta-analysis | Method that compares 3+ interventions simultaneously in a single analysis by combining results from direct comparisons and indirect comparisons across a network of primary studies. Produces relative effects for all pairs of interventions in the network and yields more precise estimates than a single direct or indirect estimate. Can be conducted within a systematic review. | Aims to be exhaustive and comprehensive | Risk of bias performed for included studies | Graphical, tabular, and rankograms of interventions with narrative commentary. Forest plot can be used to present summary relative effects for pairwise comparisons. | Summary statistics of intervention effects for all pairs of interventions as well as measures of incoherence and heterogeneity |
| Overview of reviews (also called “Umbrella review” or “review of reviews”) | Compilation of evidence from multiple systematic reviews into one document. May investigate the same intervention for different conditions, problems, or populations; multiple outcomes of the same intervention for the same condition or population; or adverse effects from a single intervention or a class of related interventions for 1+ conditions. | Identification of component reviews but no search for primary studies | Includes quality or risk of bias assessment for studies within component reviews and/or reviews themselves | Graphical and tabular with narrative commentary | What is known and unknown, recommendations for practice and future research |
| Rapid review | Accelerated form of knowledge synthesis where systematic review processes are reduced or streamlined. Intended to generate evidence in a short time. | Completeness of searching determined by time/scope | Typically includes quality or risk of bias assessment, either performed by review authors or another group | Typically tabular with narrative commentary | Analysis depends on type of rapid review performed (e.g., rapid scoping review vs. rapid effectiveness review, rapid overview of reviews) |
| Scoping review | Assessment of potential size and scope of available research literature to identify the nature and extent of the evidence | Completeness of searching determined by time/scope. May include research in progress. | No quality or risk of bias assessment performed | Typically tabular with narrative commentary | Characterizes quantity and quality of literature, perhaps by study design and other key features. Attempts to specify a viable review. |

# Appendix A2) Locating existing systematic reviews

## PICO scheme

PICO stands for the following:

**P**opulation: Target population for the vaccination. Age groups, sex, ethnicity, and specific settings (e.g., hospital, school) might be specified.

**I**ntervention: Intervention being considered (e.g., vaccine formulation, doses, vaccine schedule).

**C**omparison (or absence of exposure): Refers to the alternative intervention or “action” (e.g., placebo, no vaccination, control, standard care, existing vaccine, other prevention options).

**O**utcome: This might include positive and negative (in terms of harms or adverse events) endpoints.

## Databases to search

- 1. Systematic review databases:

1. Cochrane Database of Systematic Reviews (https://www.cochranelibrary.com/cdsr/reviews)
2. Epistemonikos (https://www.epistemonikos.org/)
3. Health Evidence (https://www.healthevidence.org)
4. JBI Evidence Synthesis (formerly JBI Database of Systematic Reviews and Implementation Reports) (https://journals.lww.com/jbisrir/pages/default.aspx)
5. SAGE (https://www.who.int/immunization)

2) General bibliographic databases:

1. Embase (https://www.embase.com)
2. MEDLINE/PubMed (https://pubmed.ncbi.nlm.nih.gov/)
3. Scopus (https://www.scopus.com/search/form.uri?display=basic)
4. Turning Research into Practice (http://www.tripdatabase.com/)
5. Web of Science (https://clarivate.com/webofsciencegroup/solutions/web-of-science/)

## Devising a search strategy

Below a guidance for searching databases for SRs other than SYSVAC is provided.

1. Search strategies should include the appropriate PICO elements, e.g., terms identifying the population of interest and intervention. The Cochrane Handbook offers further information on developing search strategies [5].
2. When searching general bibliographic databases, filters designed for retrieving SRs should be used. A number of studies have published and evaluated the performance of filters for retrieving SRs [6, 7].
3. The search string should be reviewed using the Peer-Review of Search Strategies (PRESS) instrument [8], by verifying that it identifies key reviews that ought to be included, or by having it reviewed by a librarian or information specialist.

# Appendix A3) Assessing relevance and up-to-dateness

## Example for assessing relevance and up-to-dateness

Example: Harder and colleagues [9] aimed to use an existing SR to assess the efficacy/effectiveness and safety of influenza vaccination during pregnancy. Their literature search uncovered four potentially relevant SRs. The researchers assessed the relevance of the identified SRs by comparing the inclusion criteria and study characteristics of the SRs to that of their own, using tables. **Table S2**, for example, compared the PICO of the SRs. There were few differences across the SRs apart from the SR by Fell et al. (2015) [10] (see highlighted column), which did not investigate maternal outcomes or outcomes related to the efficacy or effectiveness of the vaccination in preventing influenza in the infant.

**Table S2**. Comparison of PICO (population, intervention, comparison, outcome) elements between the existing systematic review [10-13] and authors’ new review. From [9]

|  | **Galvao et al., 2013** | **Jefferson et al., 2014** | **McMillan et al., 2014** | **Fell et al., 2015** | **Own evidence review** |
| --- | --- | --- | --- | --- | --- |
| Population | Pregnant women and their infants | Pregnant women and their newborns | Pregnant women, their fetuses and infants up to 6 months of age | Pregnant women and their infants | Pregnant women and their infants |
| Intervention | Vaccination against influenza | Live attenuated or inactivated influenza vaccination | Inactivated influenza vaccination | Vaccination against influenza | Vaccination against seasonal influenza |
| Comparison | Placebo or other vaccines or no vaccination | Placebo or no vaccination | No vaccination | No vaccination | Placebo or no vaccination |
| Outcome | Influenza-related outcomes in mother or infant | Symptomatic influenza and influenza-like illness; maternal and pregnancy outcomes; neonatal outcomes: congenital malformations, neonatal death | Influenza, influenza-like illness, for pregnant women: adverse events and serious adverse events; for the fetus: spontaneous abortion, fetal death, premature birth, low birth weight, small for gestational age, congenital malformation | Preterm birth, early fetal death, late fetal death | Laboratory-confirmed influenza in mother and/or infant; any severe adverse event in mother or infant |

A second table (see **Table S3**) compared key study characteristics across the SRs: study design, period covered, type of vaccine (pandemic and/or seasonal), risk of bias tool(s) used, and the results of the risk of bias assessment.

**Table S3**. Comparison of study characteristics between the existing systematic reviews [10-13] and authors’ new review. From [9]

|  | **Galvao et al., 2013** | **Jefferson et al., 2014** | **McMillan et al., 2014** | **Fell et al., 2015** | **Own evidence review** |
| --- | --- | --- | --- | --- | --- |
| Study designs | RCTs, cohort studies | All study designs | All study designs | RCTs, cohort, cross-sectional, case–control studies | All study designs |
| Period covered | Until 09/2013 | Until 05/2013 | Until 03/2014 | Until 05/2013 | Until 03/2014 |
| Seasonal | Yes | Yes | Yes | Yes | Yes |
| Pandemic | No | Yes | Yes | Yes | No |
| Meta-analysis | No | Yes | Yes | Yes | No |
| No. of included studies | 8 | 21 | 46 | 27 | 20 |
| Risk of bias tool(s) used for RCTs | Cochrane risk of bias tool | Cochrane risk of bias tool | JBI-MASTARI | NA | Cochrane risk of bias tool |
| Risk of bias tool(s) used for observational studies | NR | NOS | JBI-MASTARI | NOS; DBC | CASP |
| Results of risk of bias assessment | NR | 10× high risk of bias; 11× unclear risk of bias | Moderate to high quality | NOS: median 8.5 (of 9) DBC: median 25 (of 31) | 9× high risk of bias; 9× low risk of bias; 2× unclear risk of bias |

CASP: Critical Appraisal Skills Program; DBC: Downs and Black Checklist; NA: not applicable; NOS: Newcastle-Ottawa-Scale; NR: not reported

The researchers then created a third table (**Table S4**) to compare the specific maternal and infant outcomes included in the four SRs under consideration.

**Table S4**. Comparison of included primary studies and outcomes in the existing systematic reviews [10-13]. From [9]

|  | **Maternal outcomes** | | | | |
| --- | --- | --- | --- | --- | --- |
| **Outcome** | **Primary study** | **Galvao et al., 2013** | **Jefferson et al., 2014** | **McMillan et al., 2014** | **Fell et al., 2015** |
| Laboratory-confirmed influenza | Thompson et al. [[30](https://systematicreviewsjournal.biomedcentral.com/articles/10.1186/s13643-016-0347-9#ref-CR30)] | No (unclear) | No (date) | Yes | No (criteria) |
|  | Zaman et al. [[31](https://systematicreviewsjournal.biomedcentral.com/articles/10.1186/s13643-016-0347-9#ref-CR31)] | No (unclear) | No (unclear) | Yes | No (criteria) |
| Influenza-like illness | Black et al. [[32](https://systematicreviewsjournal.biomedcentral.com/articles/10.1186/s13643-016-0347-9#ref-CR32)] | No (unclear) | Yes | Yes | No (criteria) |
|  | Hulka [[33](https://systematicreviewsjournal.biomedcentral.com/articles/10.1186/s13643-016-0347-9#ref-CR33)] | Yes | Yes | No (unclear) | No (criteria) |
|  | Munoz et al. [[34](https://systematicreviewsjournal.biomedcentral.com/articles/10.1186/s13643-016-0347-9#ref-CR34)] | No (unclear) | No (unclear) | Yes | No (criteria) |
|  | Zaman et al. [[31](https://systematicreviewsjournal.biomedcentral.com/articles/10.1186/s13643-016-0347-9#ref-CR31)] | Yes | No (unclear) | No (unclear) | No (criteria) |
| Local adverse events | Hulka [[33](https://systematicreviewsjournal.biomedcentral.com/articles/10.1186/s13643-016-0347-9#ref-CR33)] | No (criteria) | No (unclear) | Yes | No (criteria) |
|  | Yeager et al. [[35](https://systematicreviewsjournal.biomedcentral.com/articles/10.1186/s13643-016-0347-9#ref-CR35)] | No (criteria) | No (unclear) | Yes | No (criteria) |
|  | Zaman et al. [[31](https://systematicreviewsjournal.biomedcentral.com/articles/10.1186/s13643-016-0347-9#ref-CR31)] | No (criteria) | No (unclear) | Yes | No (criteria) |
| Systemic adverse events | Englund et al. [[36](https://systematicreviewsjournal.biomedcentral.com/articles/10.1186/s13643-016-0347-9#ref-CR36)] | No (criteria) | No (unclear) | Yes | No (criteria) |
|  | Hulka [[33](https://systematicreviewsjournal.biomedcentral.com/articles/10.1186/s13643-016-0347-9#ref-CR33)] | No (criteria) | No (unclear) | Yes | No (criteria) |
|  | Lin et al. [[37](https://systematicreviewsjournal.biomedcentral.com/articles/10.1186/s13643-016-0347-9#ref-CR37)] | No (criteria) | No (unclear) | Yes | No (criteria) |
|  | Yeager et al. [[35](https://systematicreviewsjournal.biomedcentral.com/articles/10.1186/s13643-016-0347-9#ref-CR35)] | No (criteria) | No (unclear) | Yes | No (criteria) |
|  | Zaman et al. [[31](https://systematicreviewsjournal.biomedcentral.com/articles/10.1186/s13643-016-0347-9#ref-CR31)] | No (criteria) | No (unclear) | Yes | No (criteria) |
| Serious adverse events | Munoz et al. [[34](https://systematicreviewsjournal.biomedcentral.com/articles/10.1186/s13643-016-0347-9#ref-CR34)] | No (criteria) | No (unclear) | Yes | No (criteria) |
|  | Nordin et al. [[38](https://systematicreviewsjournal.biomedcentral.com/articles/10.1186/s13643-016-0347-9#ref-CR38)] | No (criteria) | Yes | Yes | No (criteria) |
| Preeclampsia | Munoz et al. [[34](https://systematicreviewsjournal.biomedcentral.com/articles/10.1186/s13643-016-0347-9#ref-CR34)] | No (criteria) | No (unclear) | Yes | No (criteria) |

|  | **Infant outcomes** | | | | |
| --- | --- | --- | --- | --- | --- |
| **Outcome** | **Primary study** | **Galvao et al., 2013** | **Jefferson et al., 2014** | **McMillan et al., 2014** | **Fell et al., 2015** |
| Lab-confirmed influenza | Benowitz et al. [[39](https://systematicreviewsjournal.biomedcentral.com/articles/10.1186/s13643-016-0347-9#ref-CR39)] | No (criteria) | Yes | Yes | No (criteria) |
|  | Eick et al. [[40](https://systematicreviewsjournal.biomedcentral.com/articles/10.1186/s13643-016-0347-9#ref-CR40)] | Yes | Yes | Yes | No (criteria) |
|  | Poeling et al. [[41](https://systematicreviewsjournal.biomedcentral.com/articles/10.1186/s13643-016-0347-9#ref-CR41)] | No (criteria) | Yes | Yes | No (criteria) |
|  | Zaman et al. [[31](https://systematicreviewsjournal.biomedcentral.com/articles/10.1186/s13643-016-0347-9#ref-CR31)] | Yes | No (unclear) | Yes | No (criteria) |
| Influenza-like illness | Black et al. [[32](https://systematicreviewsjournal.biomedcentral.com/articles/10.1186/s13643-016-0347-9#ref-CR32)] | No (unclear) | Yes | Yes | No (criteria) |
|  | Eick et al. [[40](https://systematicreviewsjournal.biomedcentral.com/articles/10.1186/s13643-016-0347-9#ref-CR40)] | No (unclear) | Yes | Yes | No (criteria) |
|  | France et al. [[42](https://systematicreviewsjournal.biomedcentral.com/articles/10.1186/s13643-016-0347-9#ref-CR42)] | No (unclear) | Yes | No (unclear) | No (criteria) |
|  | Munoz et al. [[34](https://systematicreviewsjournal.biomedcentral.com/articles/10.1186/s13643-016-0347-9#ref-CR34)] | No (unclear) | No (unclear) | Yes | No (criteria) |
|  | Zaman et al. [[31](https://systematicreviewsjournal.biomedcentral.com/articles/10.1186/s13643-016-0347-9#ref-CR31)] | Yes | No (unclear) | Yes | No (criteria) |
| Premature birth (<37 weeks) | Black et al. [[32](https://systematicreviewsjournal.biomedcentral.com/articles/10.1186/s13643-016-0347-9#ref-CR32)] | Yes | Yes | Yes | Yes |
|  | Chambers et al. [[43](https://systematicreviewsjournal.biomedcentral.com/articles/10.1186/s13643-016-0347-9#ref-CR43)] | No (unclear) | No (date) | Yes | Yes |
|  | Dodds et al. [[44](https://systematicreviewsjournal.biomedcentral.com/articles/10.1186/s13643-016-0347-9#ref-CR44)] | No (unclear) | No (unclear) | No (unclear) | Yes |
|  | Legge et al. [[45](https://systematicreviewsjournal.biomedcentral.com/articles/10.1186/s13643-016-0347-9#ref-CR45)] | No (date) | No (date) | No (unclear) | Yes |
|  | Louik et al. [[46](https://systematicreviewsjournal.biomedcentral.com/articles/10.1186/s13643-016-0347-9#ref-CR46)] | No (date) | No (unclear) | No (unclear) | Yes |
|  | Munoz et al. [[34](https://systematicreviewsjournal.biomedcentral.com/articles/10.1186/s13643-016-0347-9#ref-CR34)] | Yes | Yes | Yes | Yes |
|  | Omer et al. [[47](https://systematicreviewsjournal.biomedcentral.com/articles/10.1186/s13643-016-0347-9#ref-CR47)] | Yes | Yes | Yes | Yes |
|  | Sheffield et al. [[48](https://systematicreviewsjournal.biomedcentral.com/articles/10.1186/s13643-016-0347-9#ref-CR48)] | Yes | Yes | Yes | Yes |
|  | Steinhoff et al. [[49](https://systematicreviewsjournal.biomedcentral.com/articles/10.1186/s13643-016-0347-9#ref-CR49)] | No (unclear) | No (unclear) | No (unclear) | Yes |
|  | Zaman et al. [[31](https://systematicreviewsjournal.biomedcentral.com/articles/10.1186/s13643-016-0347-9#ref-CR31)] | Yes | No (unclear) | No (unclear) | No (unclear) |
| Fetal death (>500 g) | Sheffield et al. [[48](https://systematicreviewsjournal.biomedcentral.com/articles/10.1186/s13643-016-0347-9#ref-CR48)] | Yes | Yes | Yes | Yes |
| Spontaneous abortion | Irving et al. [[50](https://systematicreviewsjournal.biomedcentral.com/articles/10.1186/s13643-016-0347-9#ref-CR50)] | No (criteria) | No (date) | Yes | No (criteria) |
| Congenital malformation | Munoz et al. [[34](https://systematicreviewsjournal.biomedcentral.com/articles/10.1186/s13643-016-0347-9#ref-CR34)] | No (criteria) | Yes | No (unclear) | No (criteria) |
|  | Sheffield et al. [[48](https://systematicreviewsjournal.biomedcentral.com/articles/10.1186/s13643-016-0347-9#ref-CR48)] | No (criteria) | Yes | Yes | No (criteria) |
| Small for gestational age | Omer et al. [[47](https://systematicreviewsjournal.biomedcentral.com/articles/10.1186/s13643-016-0347-9#ref-CR47)] | Yes | No (unclear) | Yes | No (criteria) |
|  | Sheffield et al. [[48](https://systematicreviewsjournal.biomedcentral.com/articles/10.1186/s13643-016-0347-9#ref-CR48)] | Yes | No (unclear) | Yes | No (criteria) |
|  | Zaman et al. [[31](https://systematicreviewsjournal.biomedcentral.com/articles/10.1186/s13643-016-0347-9#ref-CR31)] | Yes | No (unclear) | No (unclear) | No (criteria) |
| Neonatal death | Sheffield et al. [[48](https://systematicreviewsjournal.biomedcentral.com/articles/10.1186/s13643-016-0347-9#ref-CR48)] | Yes | Yes | No (unclear) | No (criteria) |

No: not included (with reasons for exclusion in parenthesis: date = published after search date of the systematic review; criteria = inclusion criteria of the systematic review not met; unclear = reason for exclusion from the systematic review unclear); Yes: included

Based on these analyses, Harder et al. (2016) [9] identified the SR by McMillan et al. (2014) [13] as the most promising to use for two reasons: (1) it closely matched the PICO and study characteristics of their own evidence review, and (2) it was the most comprehensive of the SRs, as it included the highest number of studies and outcomes.

# Appendix A4) Assessing methodological quality

## Example for assessing methodological quality of a systematic review with AMSTAR 2

Example: **Table S5** presents the results from applying AMSTAR 2 to a SR [14].

Highlighted rows represent the domains that the developers of AMSTAR 2 generally regarded as “critical” [15]. They acknowledged, however, that these domains will not always be “critical,” for example, if a review only contains high quality randomized controlled trials, a known literature base is being summarized using meta-analysis, or if meta-analysis was not performed and suggest that appraisers add or substitute critical domains as they see fit [15].

**Table S5**. Application of AMSTAR 2 of a systematic review [14]. Orange boxes represent proposed critical items by [15].

| **AMSTAR 2 Item** | **Assessment** | **Explanation** | **Example from text** |
| --- | --- | --- | --- |
| **1. Did the research questions and inclusion criteria for the review include the components of PICO?** | Yes | Population, Intervention, Comparison, and Outcome all stated | Population: “HIV-infected or HEU children aged 0-18 years”  Intervention: “Studies were eligible for inclusion in the systematic review if they reported in immunogenicity or safety of any measles vaccination strategy”  “For inclusion in the immunogenicity meta-analysis, studies needed to report on primary or booster vaccination”  Comparison: “a comparator group of either HIV-uninfected children (HEU/HIV-unexposed) or HIV-infected children on a different antiretroviral therapy (ART) regimen”  Outcome: “The outcomes of interest were immunogenicity and safety. Immunogenicity: studies were included if data were reported as proportions of subjects with seroprotective (≥330 MIU/mL or as indicated by authors), seropositive, or seroconversion (4-fold rise in titre or change from seronegative or seropositive) measles antibody responses. A composte outcome for seroresponse was created using seroprotection rates post-vaccination, and if not available, seropositivity or seroconversion rates were considered. Safety: all reported safety outcomes post-vaccination were considered, including deaths, severe adverse events (SAEs) other than death and adverse events (AEs).” |
| **2. Did the report of the review contain an explicit statement that the review methods were established prior to the conduct of the review and did the report justify and significant deviations from the protocol?** | Yes | PROSPERO number given, which links to a full record on PROSPERO with all methods, registered before the start of review | “PROSPERO registration number: CRD42017057411” |
| **3. Did the review authors explain their selection of the study designs for inclusion in the review?** | Yes | Explanation for including both RCTs and NRSI given | “Eligible study designs were interventional or observational. For assessment of safety, case reports were also included.” |
| **4. Did the review authors use a comprehensive literature search strategy?** | Yes | Searched multiple databases, provided strategy, justified restrictions, searched reference lists, searched trial registries, included experts in the field, searched for grey literature and conducted search within 24 months of completion of the review | Methods section 2.1 and Flow chart in section 3. |
| **5. Did the review authors perform study selection in duplicate?** | Yes | At least two reviewers independently agreed on selection of eligible studies and achieved consensus on which studies to include | “Two independent reviewers (EM, MvR) screened titles and abstracts of identified studies. Articles were retained if they met the inclusion criteria according to one or both of the reviewers.” |
| **6. Did the review authors perform data extraction in duplicate?** | No | No mention of achieved consensus or agreement between both data extractors | “Data were extracted from manuscripts”  “EM and MvR performed screening and data extraction” |
| **7. Did the review authors provide a list of excluded studies and justify the exclusions?** | Yes | Provided a list of all potentially relevant studies that were read in full text form but excluded, and justified the exclusion from the review | Fig 1. Flow chart of study selection |
| **8. Did the review authors describe included studies in adequate detail?** | Yes | Described PICO, population in detail, intervention and comparator in detail, study setting, and timeframe for follow-up | Table 1, Supplementary data 4 |
| **9. Did the review authors use a satisfactory technique for assessing the risk of bias (RoB) in individual studies that were included in the review?** | Yes | Assessed RoB of included RCTs and NRSIs from all relevant potential RoBs. | Fig 5., Supplementary data 3 and 10 |
| **10. Did the review authors report on the sources of funding for the studies included in the review?** | No | Did not report on the sources of funding for individual studies included in the review, and no mention that the reviewers looked for this information | - |
| **11. If meta-analysis was performed, did the review authors use appropriate methods for statistical combination of results?** | Yes | The authors justified combining the data in a meta-analysis and used an appropriate weighted technique to combine study results and adjusted for/investigated the causes of heterogeneity | “…using risk ratios and 99%Cis stratified by vaccination dose and age at vaccination. In case of significant heterogeneity, a random-effects model was applied. To explore statistical variation and heterogeneity between trials, pre-specified subgroup analysis were performed based on outcome (seroprotection, serological test, use of ART, study design, age of vaccination and time interval between vaccination and measurement of serological response.” |
| **12. If meta-analysis was performed, did the review authors assess the potential impact of RoB in individual studies on the results of the meta-analysis or other evidence synthesis?** | Yes | The authors performed analyses to investigate possible impact of RoB on summary estimates of effect | “Meta-regression was used to explore between-study variance not explained by the covariates” |
| **13. Did the review authors account for RoB in primary studies when interpreting/discussing the results of the review?** | Yes | The review provided a discussion of the likely impact of RoB on the results | “Our results need to be interpreted in the context of the risk of bias evaluation and low to very low quality of evidence. All studies included in this review were of observational nature, except for one RCT. Observational studies may be subject to selection and confounding bias.” |
| **14. Did the review authors provide a satisfactory explanation for, and discussion of, any heterogeneity observed in the results of the review?** | Yes | Authors performed an investigation of sources of heterogeneity in the results and discussed the impact of this on the results of the review | “This might be due to selection of HIV-infected children that survived to an older age, who were likely to be slow progressors and maintained their immunological status, or received ART.”  “This could be explained by reduced transplacental transfer of antibodies from HIV-infected women, resulting in lower levels of maternal antibodies in the infant and less interference with the B-cell response to vaccination. Maternal PMTCT regiments and breastfeeding recommendations for HIV-infected mothers varied substantially between 1987 and 2018, and may have contributed to differences between HEU and other groups.”  “due to absence of direct comparisons between vaccinated and unvaccinated HIV-infected children and poor quality of reporting, limited conclusions can be drawn from this analysis. HIV-infected children may experience more SAEs due to their underlying illness, unrelated to vaccine administration”  “Our results need to be interpreted in the context of the risk of bias evaluation and low to very low quality of evidence…observational studies may be subject to selection and confounding bias” |
| **15. If they performed quantitative synthesis did the review authors carry out an adequate investigation of publication bias (small study bias) and discuss its likely impact on the results of the review?** | Yes | Performed tests for publication bias and discussed the likelihood and magnitude of impact of publication bias | “risk of publication bias was assessed using normal and contour-enhanced funnel plots if ten or more articles were included in the meta-analysis. Small study effects were evaluated using Egger’s-test for asymmetry.” |
| **16. Did the review authors report any potential sources of conflict of interest, including any funding they received for conducting the review?** | Yes | The authors described their funding sources and how they managed potential conflicts of interest | “The funder of the study had no role in study design, data collection, data analysis, data interpretation, or writing of the report.”  “This work was partially supported by the South African Research Chairs Initative of the Department of Science and Technology, National Research Foundation in Vaccine Preventable Diseases (MADH018), and the Medical Research Counter” … “MCN reports personal fees from Pfizer and non-financial support from Sanofi outside submitted work. SAM reports grants from Medical Research Council South Africa, grants from Department Science and Technology/National Research Foundation during the conduct of the study; grants and personal fees from the Bill and Melinda Gates Foundation, grants from GSK, grants and personal fees from Sanofi, grants from Pfizer outside the submitted work.” |
| **Overall confidence in review results:** | Moderate | More than 1 non-critical weakness: #6 (Data extraction in duplicate) and #10 (Reporting on sources of funding for studies included in the review) |  |

PROSPERO: International prospective register of systematic reviews; RoB: risk of bias

## Reasons for updating systematic reviews


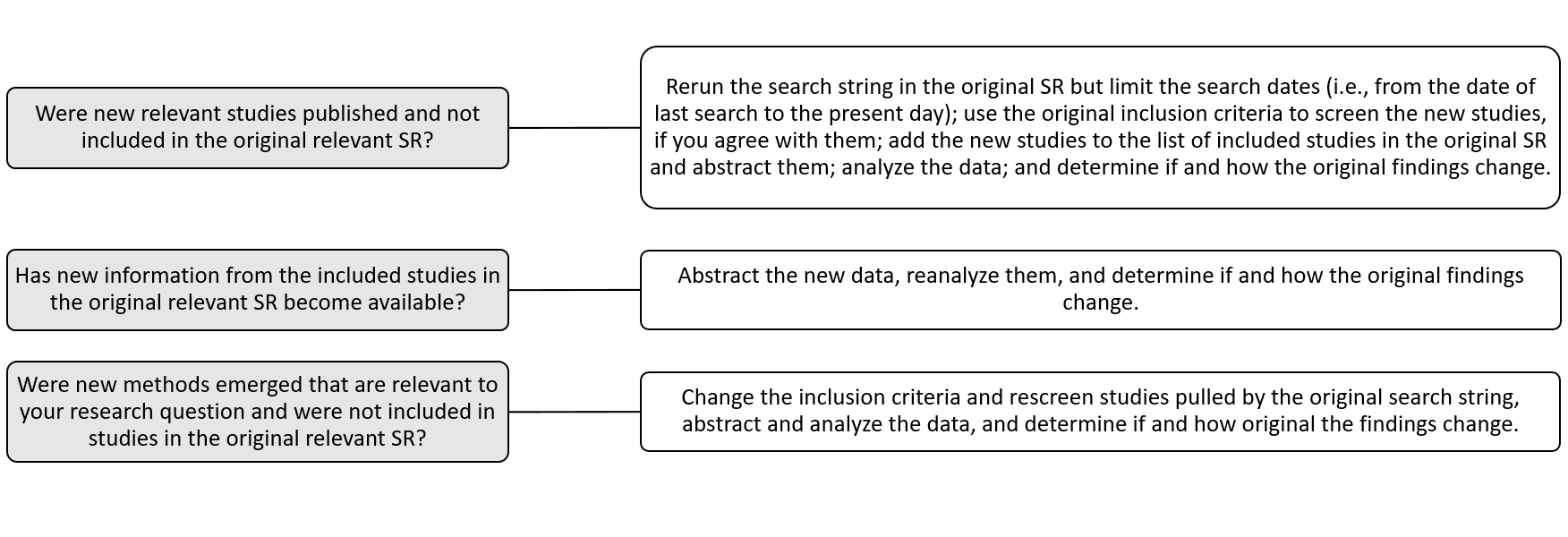

**Figure S1**. Reasons for updating systematic reviews
SR: systematic review

# References

1. Grant MJ, Booth A. A typology of reviews: an analysis of 14 review types and associated methodologies. Health Info Libr J. 2009;26(2):91-108. doi: <https://doi.org/10.1111/j.1471-1842.2009.00848.x>.

2. Tricco AC, Langlois EV, Straus editors SE. Rapid reviews to strengthen health policy and systems: a practical guide. Geneva: World Health Organization. 2017. doi: <http://www.who.int/alliance-hpsr/resources/publications/rapid-review-guide/en>.

3. Chaimani A, Caldwell DM, Li T, Higgins JPT, Salanti G. Chapter 11: Undertaking network meta-analyses. In: Higgins JPT, Thomas J, Chandler J, Cumpston M, Li T, Page MJ, et al., editors. Cochrane Handbook for Systematic Reviews of Interventions version 62 (updated February 2021): Cochrane; 2021.

4. Editorial Decision Tree for Overviews. Cochrane Methods Comparing Multiple Interventions n.d. Available from: <https://methods.cochrane.org/sites/methods.cochrane.org.cmi/files/public/uploads/DecisionChart.pdf>

5. Lefebvre C, Glanville J, Briscoe S, Littlewood A, Marshall C, Metzendorf M-I, et al. Chapter 4: Searching for and selecting studies. In: Higgins JPT, Thomas J, Chandler J, Cumpston M, Li T, Page MJ, et al., editors. Cochrane Handbook for Systematic Reviews of Interventions version 60 (updated July 2019): Cochrane; 2019.

6. Glanville J, Lefebvre C, Manson P, Robinson S, Shaw N, editors. ISSG Search Filter Resource York (UK): The InterTASC Information Specialists' Sub-Group; 2006 [updated 4 June 2021]. Available from: <https://sites.google.com/a/york.ac.uk/issg-search-filters-resource/home>

7. Lunny C, Brennan SE, McDonald S, McKenzie JE. Toward a comprehensive evidence map of overview of systematic review methods: paper 1-purpose, eligibility, search and data extraction. Syst Rev. 2017;6(1):231. doi: <https://doi.org/10.1186/s13643-017-0617-1>.

8. McGowan J, Sampson M, Salzwedel DM, Cogo E, Foerster V, Lefebvre C. PRESS Peer Review of Electronic Search Strategies: 2015 Guideline Statement. J Clin Epidemiol. 2016;75:40-6. doi: <https://doi.org/10.1016/j.jclinepi.2016.01.021>.

9. Harder T, Remschmidt C, Haller S, Eckmanns T, Wichmann O. Use of existing systematic reviews for evidence assessments in infectious disease prevention: a comparative case study. Syst Rev. 2016;5(1):171. doi: <https://doi.org/10.1186/s13643-016-0347-9>.

10. Fell DB, Platt RW, Lanes A, Wilson K, Kaufman JS, Basso O, et al. Fetal death and preterm birth associated with maternal influenza vaccination: systematic review. BJOG. 2015;122(1):17-26. doi: <https://doi.org/10.1111/1471-0528.12977>.

11. Demicheli V, Jefferson T, Al-Ansary LA, Ferroni E, Rivetti A, Di Pietrantonj C. Vaccines for preventing influenza in healthy adults. Cochrane Database Syst Rev. 2014;(3). doi: <https://doi.org/10.1002/14651858.CD001269.pub5>.

12. Galvao TF, Silva MT, Zimmermann IR, Lopes LA, Bernardo EF, Pereira MG. Influenza vaccination in pregnant women: a systematic review. ISRN Prev Med. 2013;2013:879493. doi: <https://doi.org/10.5402/2013/879493>.

13. McMillan M, Kralik D, Porritt K, Marshall H. Influenza vaccination during pregnancy: a systematic review of effectiveness and adverse events. The JBI Database of Systematic Reviews and Implementation Reports. 2014;Vol 12(No 6 ).

14. Mutsaerts E, Nunes MC, van Rijswijk MN, Klipstein-Grobusch K, Grobbee DE, Madhi SA. Safety and Immunogenicity of Measles Vaccination in HIV-Infected and HIV-Exposed Uninfected Children: A Systematic Review and Meta-Analysis. EClinicalMedicine. 2018;1:28-42. doi: <https://doi.org/10.1016/j.eclinm.2018.06.002>.

15. Shea BJ, Reeves BC, Wells G, Thuku M, Hamel C, Moran J, et al. AMSTAR 2: a critical appraisal tool for systematic reviews that include randomised or non-randomised studies of healthcare interventions, or both. BMJ. 2017;358:j4008. doi: <https://doi.org/10.1136/bmj.j4008>.
